# Special Issue—Immunity to Influenza Viruses

**DOI:** 10.3390/v14020319

**Published:** 2022-02-03

**Authors:** Marios Koutsakos, Sophie A. Valkenburg

**Affiliations:** 1 Department of Microbiology and Immunology, Peter Doherty Institute for Infection and Immunity, University of Melbourne, Melbourne, VIC 3000, Australia; 2 HKU Pasteur Research Pole, School of Public Health, The University of Hong Kong, Hong Kong, China

Influenza viruses remain a constant global threat with significant health and socioeconomic impact every year and have the potential to cause devastating pandemics. Despite this, current vaccines against influenza viruses are only modestly effective, require annual reformulation, and may not provide protection against emerging influenza viruses from animal reservoirs. Therefore, new vaccines are needed to combat influenza viruses. The design of such vaccines requires a thorough understanding of the immune response to influenza viruses and of the host–pathogen interactions that determine immune disease versus immune protection. Therefore, the aim of this Special Issue on “Immunity to Influenza Viruses” is to explore the immune response to influenza viruses from humans and animals.

The innate immune system forms the first line of defense against viral infections, priming the adaptive response, and has a critical role in controlling influenza viruses. Most critically and the least well understood is local innate mucosal immunity at the respiratory tract. Mifsud, Kuba, and Barr [1] provide an elaborate summary of innate immune responses in the upper and lower respiratory tract and the crucial balance between controlling influenza virus replication and immunopathology.

Regarding the adaptive immune response to infection and vaccination, both humoral and cellular immune responses are covered in this Special Issue, with a focus on factors that influence the generation B and T cell memory, as well as the approaches available to study immunity to influenza viruses. Guthmiller, Utset, and Wilson [2] discuss the generation of B cell memory against antigenically diverse influenza viruses, the factors that influence the generation of broadly neutralizing antibodies, and how innovative vaccine strategies can be utilized to induce such broadly protective immunity. Schmidt and Lapuente [3] summarize the literature on tissue-resident memory T cell responses to influenza viruses, strategies for eliciting such responses by vaccination, as well as the challenges faced in inducing and assessing tissue-resident memory T cell responses in humans. Tomic, Pollard, and Davis [4] provide a thorough overview of systems' immunology approaches and their role in dissecting immune correlates of protection for influenza to determine the immune interactions for effective viral control and vaccine efficacy. Lin et al. [5] focus on the issue of antibody non-responsiveness to influenza infection and vaccination, discussing biological factors that may impact antibody responses and technical factors that may influence their analysis. Bull et al. [6] discuss the potential impacts of next-generation vaccines that may be non-sterilizing, leading to increased immune evasion by rapidly mutating influenza viruses. Sekiya et al. [7] discuss the merits and pitfalls of currently approved and novel vaccine strategies against influenza viruses.

Anti-influenza immunity conferred by vaccination is further assessed in three original research publications. Mital et al. [8] determine the protective capacity of recombinant influenza HA ectodomain fusion proteins against H1N1 and H3N2 challenge in mice. Mytle et al. [9] demonstrate the protective capacity of MVA vector-encoded nucleoprotein (NP) and M2 ectodomain against the H1N1 and H5N1 challenge of mice. Otani et al. [10] assess IFNγ and GrzB responses following in vitro restimulation of peripheral blood mononuclear cells prior to and following vaccination of healthy human donors, and the relationship of these measurements to the antibody response following vaccination.

In addition to immune responses in humans and experimental animal models, the infection of avian hosts, such as chickens, is considered. Wang, Wei, and Liu [11] provide an overview of mucosal immunity to influenza viruses, focusing on infection of avian hosts and strategies available to mitigate influenza infections in birds. Hao et al. [12] developed a multicolor flow cytometry panel to assess cellular immunity in chickens infected with an H7N9 virus.

Overall, this Special Issue brings together unique perspectives on various aspects of influenza immunology to contribute to the field as novel vaccination strategies are developed and assessed.
